# Reversion of KPC-114 to KPC-2 in ceftazidime-avibactam- resistant/meropenem-susceptible *Klebsiella pneumoniae* ST11 is related to low mutation rates

**DOI:** 10.1128/spectrum.01173-24

**Published:** 2024-08-27

**Authors:** Jesus G. M. Pariona, Felipe Vásquez-Ponce, Johana Becerra, Thais Martins-Gonçalves, Eva M. M. Pariona, Fabio T. Madueño, Fernanda Esposito, Aline V. de Lima, Jorge L. Mello Sampaio, Rodrigo S. Galhardo, Nilton Lincopan

**Affiliations:** 1 Department of Clinical Analysis, Faculty of Pharmaceutical Sciences, Universidade de São Paulo, São Paulo, Brazil; 2 One Health Brazilian Resistance Project (OneBR), São Paulo, Brazil; 3 Department of Microbiology, Instituto de Ciências Biomédicas II, Universidade de São Paulo, São Paulo, Brazil; 4 Antimicrobial Resistance Institute of São Paulo (ARIES), São Paulo, Brazil; 5 Universidad Peruana Cayetano Heredia, Unidad de Investigación de Enfermedades Emergentes y Cambio Climático, San Martín de Porres, Peru; 6 Escola Politécnica, Engenharia Elétrica, Universidade de São Paulo, São Paulo, Brazil; Universidad de Buenos Aires, Buenos Aires, Argentina

**Keywords:** mutation rate, KPC variants, reversion mutation, meropenem susceptibility, ceftazidime-avibactam resistance, CG258

## Abstract

*Klebsiella pneumoniae* strains that produce *Klebsiella pneumoniae* Carbapenemase (KPC) variants displaying resistance to ceftazidime-avibactam (CZA) often remain susceptible to meropenem (MEM), suggesting a potential therapeutic use of this carbapenem antibiotic. However, *in vitro* studies indicate that these sorts of strains can mutate becoming MEM-resistant, raising concerns about the effectiveness of carbapenems as treatment option. We have studied mutation rates occurring from the reversion of MEM-susceptible KPC-114 to MEM-resistant KPC-2, in CZA-resistant *K. pneumoniae* belonging to ST11. Two-step fluctuation assays (FAs) were conducted. In brief, initial cultures of KPC-114-producing *K. pneumoniae* showing 1 µg/mL MEM MIC were spread on Mueller–Hinton agar plates containing 2–8 µg/mL MEM. A second step of FA, at 4–16 µg/mL MEM was performed from a mutant colony obtained at 2 µg/mL MEM. Mutation rates were calculated using maximum likelihood estimation. Parental and mutant strains were sequenced by Illumina NextSeq, and mutations were predicted by variant-calling analysis. At 8 µg/mL MEM, mutants derived from parental CZA-resistant (MIC ≥ 64 µg/mL)/MEM-susceptible (MIC = 1 µg/mL) KPC-114-positive *K. pneumoniae* exhibited an accumulative mutation rate of 3.05 × 10^−19^ mutations/cell/generation, whereas at 16 µg/mL MEM an accumulative mutation rate of 1.33 × 10^−19^ mutations/cell/generation resulted in the reversion of KPC-114 (S181_P182 deletion) to KPC-2. These findings highlight that the reversion of MEM-susceptible KPC-114 to MEM-resistant KPC-2, in CZA-resistant *K. pneumoniae* ST11 is related to low mutation rates suggesting a low risk of therapeutic failure. *In vivo* investigations are necessary to confirm the clinical potential of MEM against CZA-resistant KPC variants.

**IMPORTANCE:**

The emergence of ceftazidime-avibactam (CZA) resistance among carbapenem-resistant *Klebsiella pneumoniae* is a major concern due to the limited therapeutic options. Strikingly, KPC mutations mediating CZA resistance are generally associated with meropenem susceptibility, suggesting a potential therapeutic use of this carbapenem antibiotic. However, the reversion of meropenem-susceptible to meropenem-resistant could be expected. Therefore, knowing the mutation rate related to this genetic event is essential to estimate the potential use of meropenem against CZA-resistant KPC-producing *K. pneumoniae*. In this study, we demonstrate, *in vitro*, that under high concentrations of meropenem, reversion of KPC-114 to KPC-2 in CZA-resistant/meropenem-susceptible *K. pneumoniae* belonging to the global high-risk ST11 is related to low mutation rates.

## OBSERVATION

Ceftazidime-avibactam (CZA) therapy has significantly improved the outcomes for patients infected by carbapenem-resistant bacteria-producing *Klebsiella pneumoniae* carbapenemases (KPCs). However, resistance to CZA is a growing global concern attributed to the acquisition of mutations in KPC genes, mainly in *bla*_KPC-2_ and *bla*_KPC-3_ alleles (1–7). To date, 208 clinical variants of KPC (named KPC-2 to KPC-216) have been registered in the NCBI database [https://www.ncbi.nlm.nih.gov/pathogens/refgene/#KPC (accessed on 3 July 2024)], of which 117 have been classified as resistant to ceftazidime-avibactam-resistant (CZA-R). A total of 66 (56.4%) of these CZA-R KPC variants display activities similar to extended-spectrum β-lactamases, restoring carbapenem susceptibility. This is because carbapenems, such as meropenem (MEM) or imipenem, remain confined within the active site of KPC variants for an extended duration, functioning predominantly as an inhibitor rather than a substrate (8). Therefore, investigating the mutation rates leading to MEM resistance in global clones of *K. pneumoniae* producing KPC variants displaying an extended-spectrum beta-lactamase (ESBL) phenotype with resistance to CZA, can help predict the risk of treatment failure with MEM.

In this study, we utilized the KPC-114-positive *K. pneumoniae* strain 331 (Bioproject ID: PRJNA868780; GenBank accession number: JANTNP000000000), belonging to the international clone ST11/CG258, endemic in South America (9), as a parental strain. Derived mutants and the parental strain were submitted to comparative genomic analysis. Antimicrobial susceptibility testing was performed by disk diffusion and broth microdilution methods, with interpretative criteria based on EUCAST guidelines (10). In order to evaluate heteroresistance to MEM in strain 331, a population analysis profile (PAP) was performed (11). In this regard, strain 331 displayed resistance to CZA (MIC ≥ 64 µg/mL) and susceptibility to MEM (MIC = 1 µg/mL). Moreover, PAP analysis of this strain showed a homogenous susceptible population at subinhibitory MEM concentration (i.e., all colonies grew on media containing up to 0.5 µg/mL MEM). This methodology was devised in response to the observation of two distinct subpopulations coexisting within a single clinical isolate of KPC-producing *K. pneumoniae*, where authors reported subpopulations carrying both wild-type (WT) and polymorphic *bla*_KPC-3_ genes, conferring resistance to both carbapenem and CZA (12).

The Luria–Delbrück fluctuation assay (FA) protocol was performed in triplicates according to the instructions of Rosche and Foster (13). Twelve parallel cultures comprising 1  mL of a 2–5 × 10^3^  CFU/mL inoculum, in Mueller Hinton (MH) Broth (Oxoid), were incubated overnight at 37°C. Then two cultures were used to determine bacterial counts, and the remaining ten cultures were plated on selective media. Each entire culture was spread on MH agar containing 2–16 µg/mL MEM. Mutant colonies that grew on these plates were counted to calculate the first mutation rate (*μ*_1_), using FluCalc for MaSandri–Sarkar maximum likelihood estimation, regarded as one of the most accurate estimators available (14). A second step of FA was carried out from the mutant that grew in the first step (i.e., at 2 µg/mL MEM), with the aim of measuring the second mutation rate (*μ*_2_) leading to a high level of MEM resistance. Therefore, accumulative mutation rates (*μ*_A_) were calculated as: *μ*_A_ = *μ*_1_ × *μ*_2_ (15). The absence of mutants in FA were reported as <1 × 10^10^ mutation/cell/generation. Mutants with high levels of resistance to MEM were defined by MIC ≥ 16 µg/mL, based on EUCAST interpretative criteria (10). A cut-off value of >10^−6^ mutations per cell per generation was used to categorize a high mutation rate (16). Comparison of mutation rates was performed using the Kruskal–Wallis test and Dunn’s test as a post hoc analysis in Stata v17.0.

**FIG 1:**
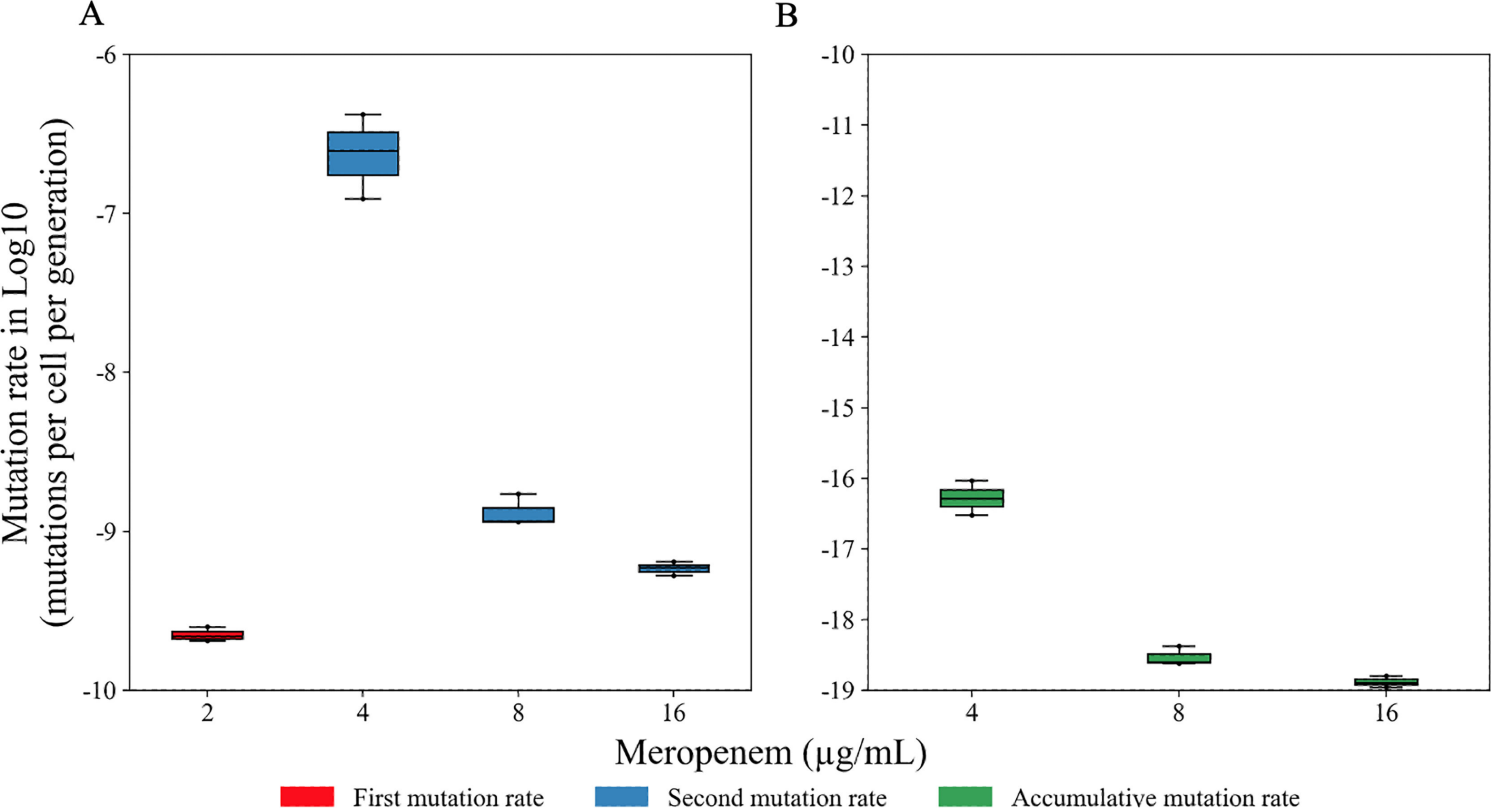
Mutation rates leading to MEM resistance in the *K. pneumoniae* strain 331 (ST11/KPC-114). In A, mutation rates obtained in the first and second steps of the FA. In B, accumulative mutation rates calculated from the first and second steps of the FA.

In the first step of FA, mean mutation rates of 2.26 × 10^−10^ mutations per cell per generation were observed for *K. pneumoniae* strain 331, when tested on MH agar plates containing 2 µg/mL MEM. In contrast, no mutants were obtained at 4 and 8 µg/mL MEM (i.e., <1 × 10^−10^ mutations per cell per generation) (Fig. 1). In the second step of FA, a single mutant colony that grew on MH agar containing 2 µg/mL MEM was selected for further investigation. This mutant was then tested on MH agar containing 4, 8, and 16 µg/mL MEM, yielding average mutation rates of 2.64 × 10^−7^, 1.34 × 10^−9^, and 5.88 × 10^−10^ mutations per cell per generation, respectively (Fig. 1). When combining the mutation rates (*μ*_A_) from the first and second steps of FA for the concentrations of 4, 8, and 16 µg/mL MEM, accumulative mutation rates of 5.88 × 10^−17^, 3.05 × 10^−19^, and 1.33 × 10^−19^ were calculated, respectively. Then, a significant difference was noted between the mutation rate obtained from selective media containing 2 µg/mL MEM and those from selective media containing 4, 8, and 16 µg/mL MEM (*P* < 0.05).

**FIG 2:**
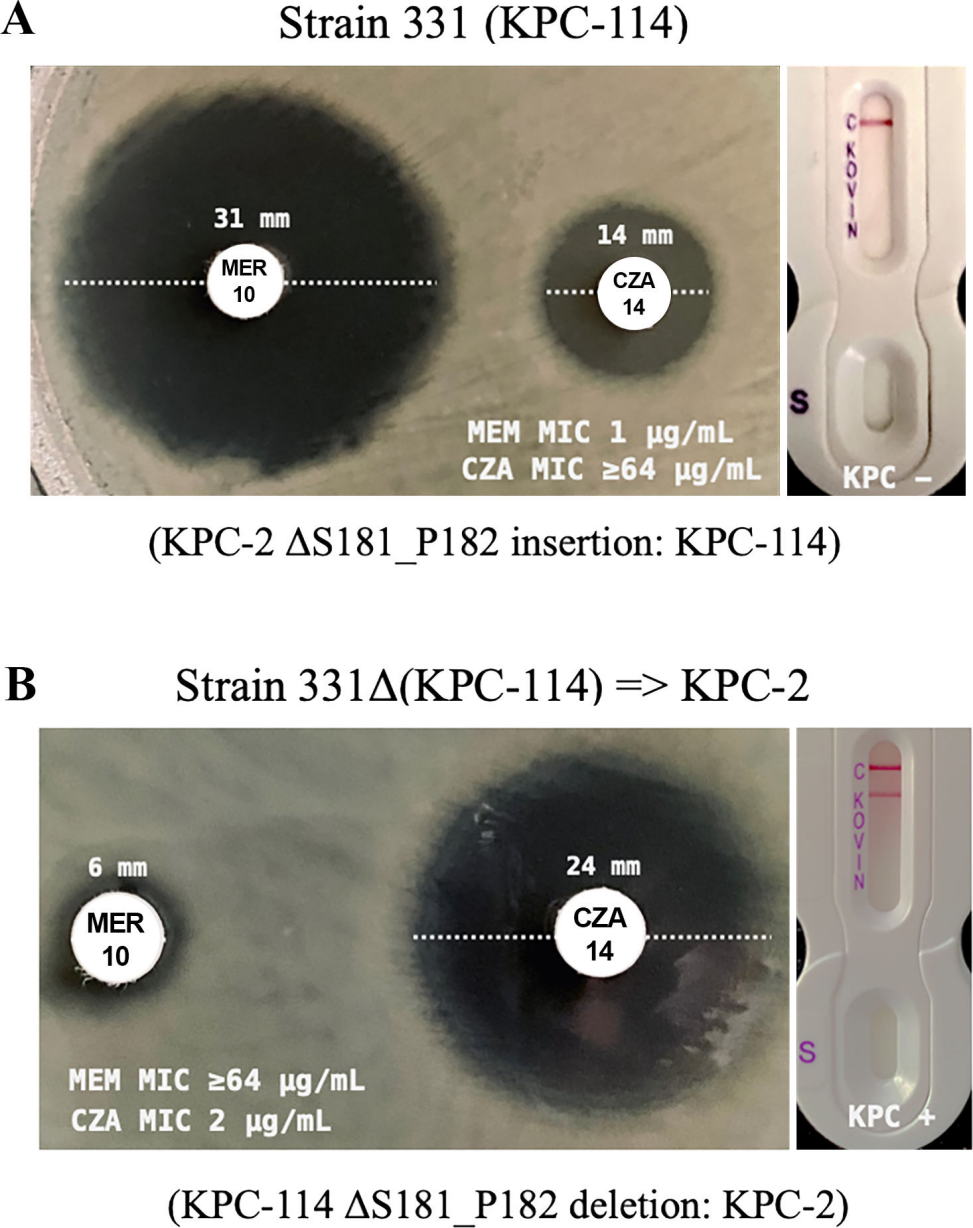
Antimicrobial susceptibility profile and NG-Test CARBA 5 lateral flow immunochromatographic of the *K. pneumoniae* strain 331 and strain 331Δ (KPC-114) ⇒ KPC-2.

Mutants grown on MH agar containing up to 4 µg/mL of MEM exhibited a low level of resistance to MEM but a high level of resistance to CZA, indicating a CZA-R and MEM-susceptible (CZA-R/MEM-S) phenotype. However, ten mutant colonies obtained from agar plates supplemented with 8 and 16 µg/mL of MEM displayed high-level MEM resistance (i.e., MICs ≥ 32 µg/mL, and inhibition zones of 6 mm) and low-level CZA resistance (i.e., MIC = 2 µg/mL, and inhibition zones of 21–24 mm) (Fig. 2). Additionally, all mutants that grew at 16 µg/mL MEM showed identical MIC values for MEM and CZA, suggesting that this specific resistance profile is associated with an accumulative mutation rate of 1.33 × 10^−19^ mutations per cell per generation. Interestingly, in contrast to the parental strain 331, the KPC enzyme was detected in mutant colonies using the NG-Test CARBA 5 lateral flow immunochromatographic assay, which is designed to identify specific carbapenemase enzymes (9).

The genomic DNA of two mutant colonies that grew in MH agar containing 16 µg/mL MEM was sequenced using the Illumina NextSeq platform (Illumina Inc., San Diego, CA, USA), and the Nextera DNA Flex library prep and 2 × 75 bp paired-end reads. Then, variant calling for NGS reads was performed using Snippy v.4.6.0 (https://github.com/tseemann/snippy). Parameters for variant calling were as follows: mutation detected in at least 75% of the reads, with a minimum coverage of 10× (17). Mutations present in regions of low coverage (two standard deviations below the average coverage of the genome) were not considered, in order to avoid variant calling in problematic mapping regions. Moreover, a visual review of the alignments using Integrative Genomics Viewer was performed in order to identify false-positive variant calls (18, 19). In this regard, an SNP-calling analysis of these two mutants confirmed reverse mutation from KPC-114 to KPC-2 (KPC-114 ΔS181_P182 deletion: KPC-2); hereafter, this mutant would be referred as strain 331Δ(KPC-114) ⇒ KPC-2 (Bioproject ID: PRJNA1100451; GenBank accession numbers: JBCEBH000000000, JBCEBI000000000) (Table S1 in the supplemental material is found at https://zenodo.org/records/13224921).

While no frameshift mutations in *omp*K35, *omp*K36, *omp*K37, and *pbp* genes were identified, one SNP in *cra* (repressor-activator for carbon metabolism; substitution Gly203Arg) and *dgaE* (D-glucosaminate-6-phosphate ammonia lyase; substitution Asp204Val) genes were predicted (Table S1). Although mutations in these genes have not been previously linked to MEM resistance, a recent study showed that in *Escherichia coli* knockout insertions in the *cra* gene can be involved in MEM tolerance (20).

A previous, *in vitro*, study described a reverse mutation in MEM-S and CZA-R, KPC-3 variant to WT KPC-3, but after a serial passage in media containing sub-inhibitory concentrations of MEM (21). Additionally, an *in vivo* study reported a reverse mutation from a KPC-33 variant to WT KPC-2 in a patient undergoing carbapenem therapy. It is worth noting that this patient initially carried a *K. pneumoniae* strain-producing WT KPC-2, raising questions about whether the reverse mutation of the KPC variant occurred during treatment or if two distinct populations (one with a KPC variant and the other with WT KPC) coexisted within the same infection site (22).

The stability of the MEM-resistant phenotype of mutants was assessed by subjecting one mutant colony to serial passage in antibiotic-free MH broth, for 10 consecutive days (23). Then MIC values were obtained by broth microdilution method, using a fresh inoculum from the 10th passage.

After stability assays, only cultures maintaining initial MEM and CZA MIC values were considered as stable mutants (23). In this regard, strain 331Δ (KPC-114) ⇒ KPC-2 remained stable with no change in categories of MEM resistance and CZA susceptibility, even after ten days of passage in an antibiotic-free medium (Table S2). This finding is consistent with a previous study, which also demonstrated the stability of mutations in KPC variants that confer resistance to CZA (24).

In Brazil, while the endemicity of ST11 has been widely documented (7), specific surveillance studies aimed at investigating the genetic basis of CZA resistance remain scarce. Therefore, data on the prevalence of KPC variants are limited (4). In this regard, recent studies from Brazil reported KPC-8, KPC-14, KPC-25, KPC-31, KPC-33, KPC-35, KPC-44, KPC-61, KPC-68, KPC-71, KPC-78, KPC-81, and KPC-90 variants; and the novel KPC-103, KPC-104, KPC-105, KPC-106, KPC-107, KPC-108, KPC-139, KPC-140, KPC141, KPC-142, and KPC-143 variants (7, 25, 26). In contrast, we have reported the emergence of KPC-113 and KPC-114 (9). The strain used in our study belongs to ST11, which is part of the international high-risk clonal group CG258, known for its wide dissemination and endemicity in South American countries (7, 27). Given this context, it is anticipated that KPC-114-producing *K. pneumoniae* ST11 may successfully disseminate in this region (9, 27).

Interestingly, most KPC variants reported in Brazil display a similar behavior to KPC114 (25, 26). In contrast, after analysis of metadata linked to KPC variants deposited in the NCBI database [https://www.ncbi.nlm.nih.gov/pathogens/refgene/#KPC (accessed on 3 July 2024)], we confirmed that among 117 KPC variants deposited as CZA-resistant, so far, 66 (56.4%) display attributes similar to extended spectrum β-lactamase, remaining susceptible to MEM. Moreover, in order to confirm results presented in this study, we also evaluated a KPC-90-producing *K. pneumoniae* strain belonging to ST11, displaying a MEM-susceptible and CZA-resistant phenotype. In this regard, similar to the KPC-114-producing *K. pneumoniae*, low mutation rates (i.e., 0.9 × 10^−18^ mutations/cell/generation) for the reversion of MEM-susceptible/CZA-resistant KPC-90 variant to the WT MEM-resistant/CZA-susceptible KPC-2, were observed.

Finally, although the MEM-susceptible *K. pneumoniae* strain 331 carried the *bla*_KPC-114_ variant on an IncN plasmid, repeated conjugation assays between the clinical KPC-114 isolate and *E. coli* DH5α or *E. coli* C600 did not yield any transconjugants. Consequently, as a limitation, we did not include data on mutation rates of *bla*_KPC-114_-positive transconjugants. However, previous studies have shown that mutation rates in an isolate depend on a combination of factors, including bacterial species, plasmid characteristics, and plasmid copy numbers (15, 28). Conjugation might significantly alter these factors, potentially affecting mutation rates drastically (15). Notably, most KPC variants have been identified in *K. pneumoniae*, with sporadic reports in *E. coli* (https://www.ncbi.nlm.nih.gov/pathogens/isolates/#KPC). This suggests that the occurrence of these variants may be related to the bacterial species, in which the plasmid is hosted. Therefore, a more accurate estimation must be obtained by measuring the mutation rate in the original species carrying the KPC plasmid, than in a transconjugant strain belonging to a different species. In the same way, an evaluation of the mutation rate in isolates belonging to the same species and sequence type should lead to comparable results.

In summary, in this study, we demonstrate “*in vitro*” that at high concentrations of MEM, the reversion of KPC-114 to KPC-2 in CZA-resistant *K. pneumoniae* belonging to the global high-risk ST11 is associated with low mutation rates (1.33 × 10^−19^ mutations per cell per generation), where a low risk of therapeutic failure could be expected (29). In fact, in a previous study, we have shown successful MEM therapy of *Galleria mellonella* larvae infected with CZA-resistant/MEM-susceptible *K. pneumoniae* strain 331 (9). To the best of our knowledge, this study represents the first *in vitro* investigation reporting spontaneous mutation rates linked to the emergence of reversal mutations of a KPC variant to WT KPC, in a *K. pneumoniae* strain belonging to the international CG258.

## AFTER EPUB

This article was published on 27 August 2024 with name of Thais Martins-Gonçalves rendered as Thais Martins Gonçalves . The byline was corrected in the current version, posted on 4 September 2024.
